# Supplementary material for: miR-24-3p Is Antiviral Against SARS-CoV-2 by Downregulating Critical Host Entry Factors
Source: Viruses. 2024 Nov 28;16(12):1844. doi: 10.3390/v16121844 (PMC11680362; doi:10.3390/v16121844)
Supplement: Supplementary file 1 [file viruses-16-01844-s001.zip › viruses-3249093-supplementary.pdf]

Supplementary Information:

miR-24-3p Is Antiviral Against SARS-CoV-2 by Downregulating Critical Host Entry Factors

Parrish Evers [1,†], Spencer M. Uguccioni [1,†], Nadine Ahmed [1,†], Magen E. Francis [2,3], Alyson A. Kelvin [2,3] and John P. Pezacki [1,*]

[1] Department of Chemistry and Biomolecular Sciences, University of Ottawa, Ottawa, ON K1N 6N6, Canada; pever068@uottawa.ca (P.E.); suguc084@uottawa.ca (S.M.U.); nahme036@uottawa.ca (N.A.)
[2] Vaccine and Infectious Disease Organization (VIDO), University of Saskatchewan, Saskatoon, SK S7N 5E3, Canada; m.francis@usask.ca (M.E.F.); alyson.kelvin@usask.ca (A.A.K.)
[3] Department of Biochemistry, Microbiology, and Immunology, University of Saskatchewan, Saskatoon, SK S7N 5E3, Canada
* Correspondence: john.pezacki@uottawa.ca
† These authors contributed equally to this work.

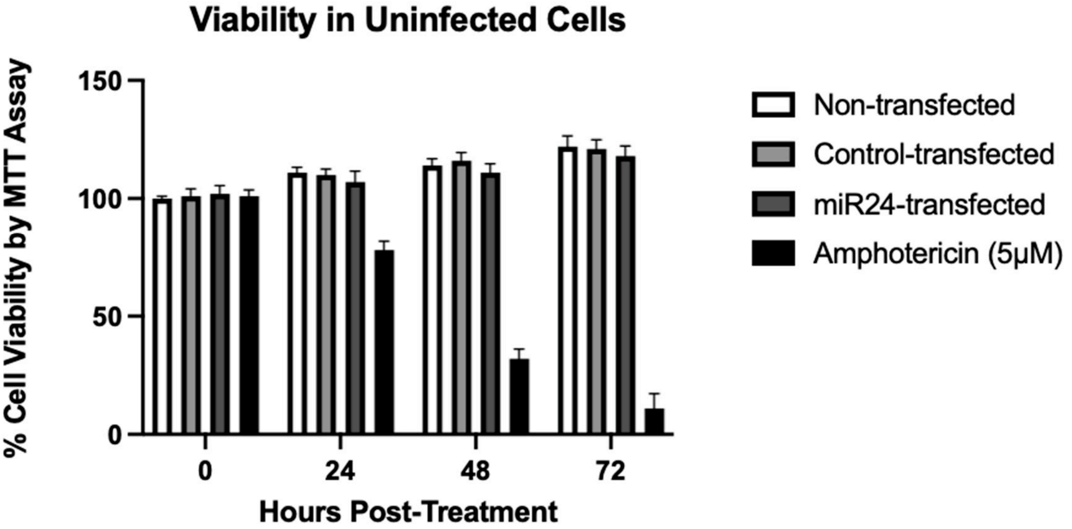

Figure S1. MTT Calu-3 cell viability assay during transfection with miR-24. Calu-3 cells were treated with 100 nM of miR-24 or con-miR for 24-72h before incubation with 2.5 mg/mL formazan and quantifying by absorbance at 570 nm. Amphotericin was included as a positive control for cytotoxicity.

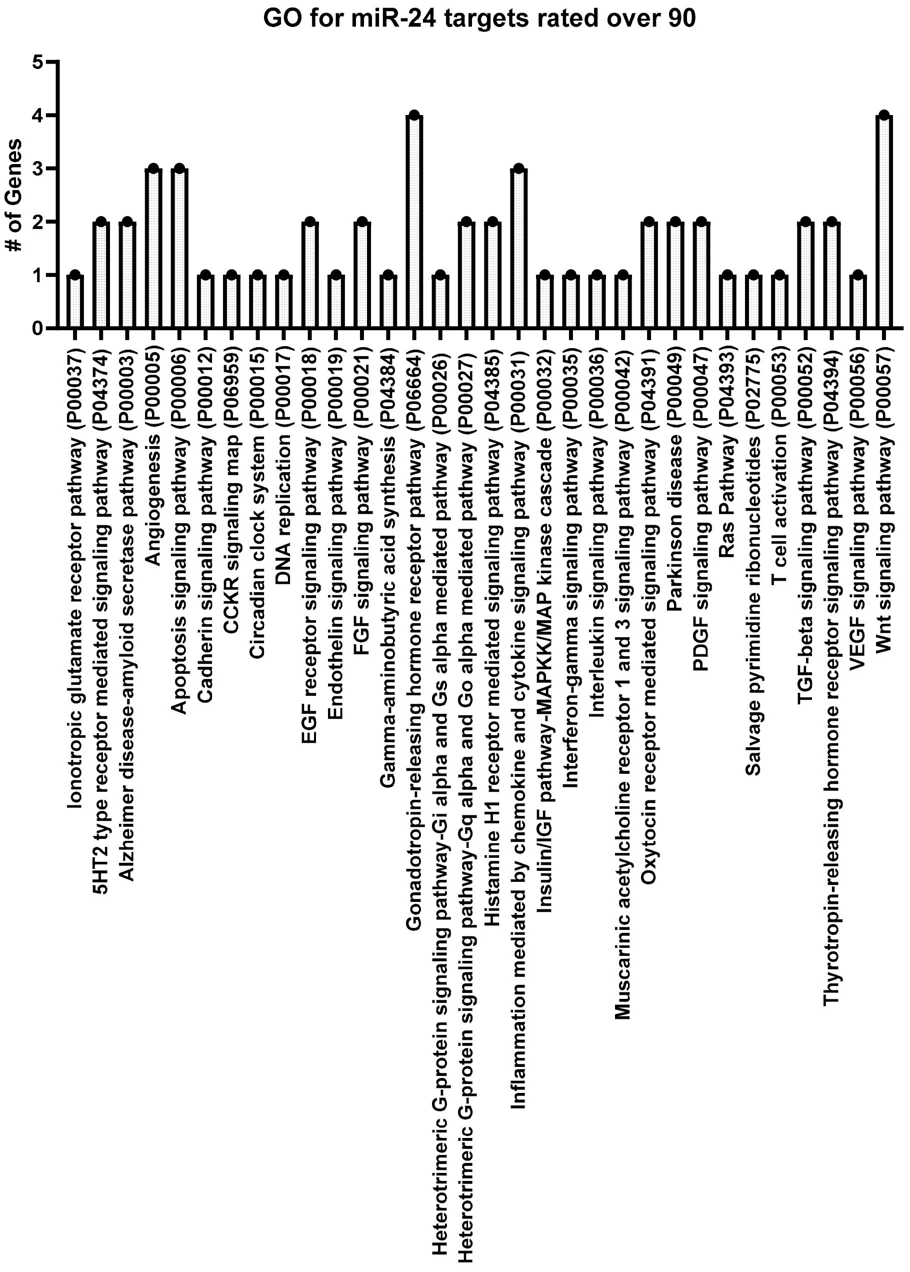

Figure S2. Gene Ontology for predicted miR-24 targets with a score of 90 of greater. Panther GO was performed on all miRDB predicted targets regardless of target expression. Most genes belonged to unclassified categories; however, these were removed for clarity. Of the 959 targets, the targets with a miRDB score of 90 or over (n = 130) were included for generating the following GO analysis.

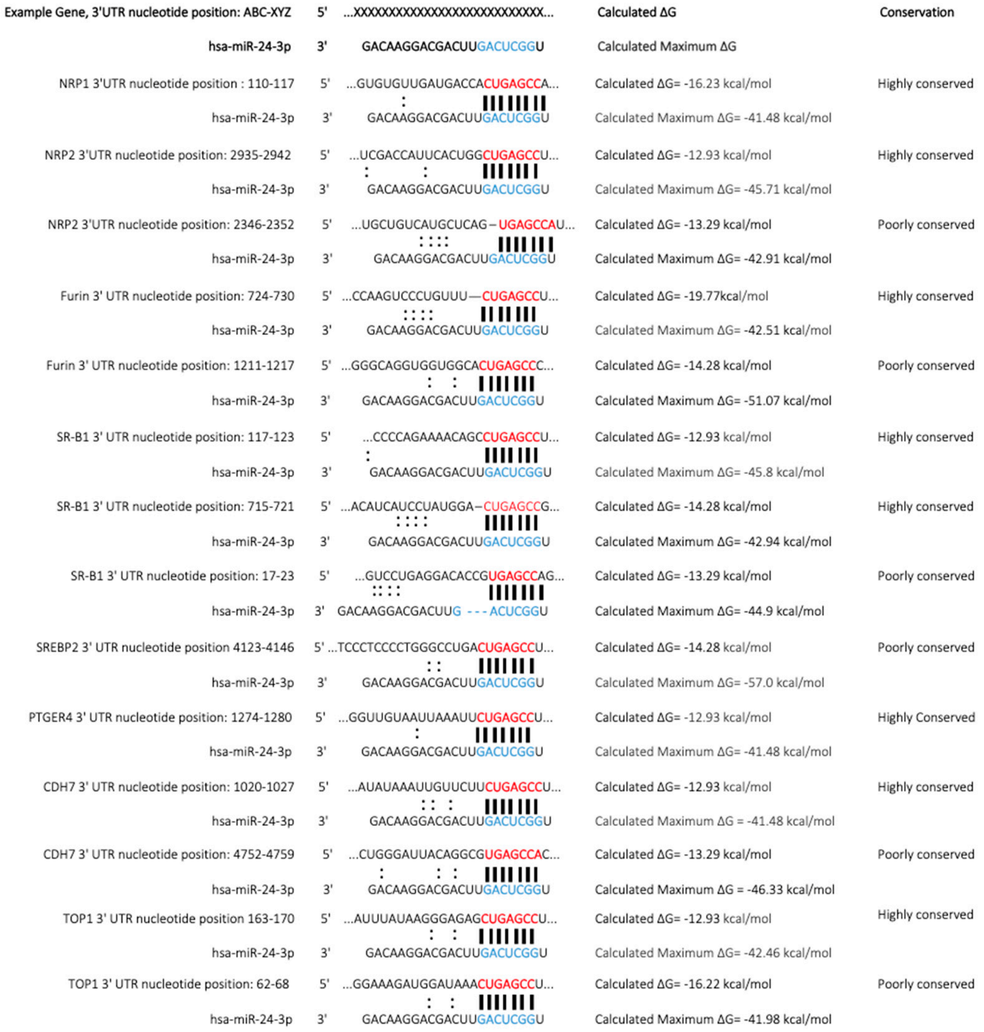

Figure S3. All 3′-UTR binding interactions and associated binding free energy for selected predicted targets. Target seed sites were predicted using miRDB [29] and additional interactions outside the seed, in addition to binding energies, were calculated/determined using Oligo Analyzer. Watson-crick pairs within the seed site are indicated by solid black lines (-) while Watson-Crick pairs and non-Watson-Crick pairs outside the seed site are indicated by a colon (:). The degree of evolutionary conservation is not part of the miRDB parameters for prediction [29]; however, the degree of conservation has been determined using Targetscan [60] and included for each target.

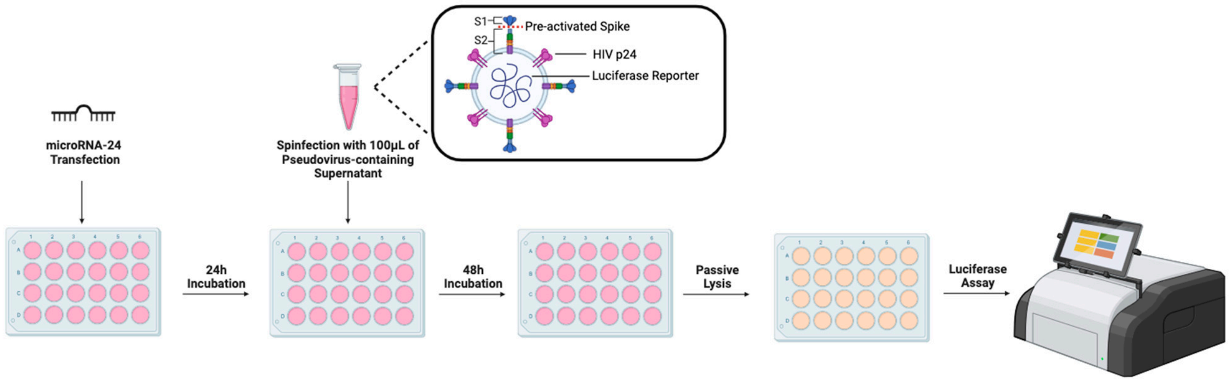

Figure S4. Scheme depicting the quantification of SARS-CoV-2 S pseudovirus entry assay.

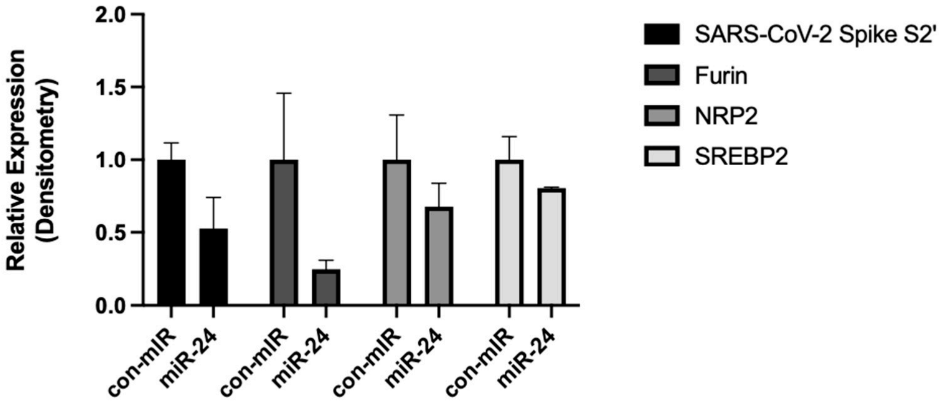

Figure S5. Densitometric analysis of western blots of miR-24 pre-treated cell lysates. HEK293T cells were pre-treated with miR-24 or con-miR 24h before transfection with the plasmids to produce pseudovirions. After 48h, the pseudovirus was collected and the pseudovirus pro-duced during miR-24 treatment or con-miR treatment were then used to infect healthy untreated Huh7 cells. A luciferase assay performed on these Huh7 cells to quantify amount of pseudovirus produced**.** The densitometry is performed on the blot shown in Figure 6B & 6C. n = 2; error bares represent SEM.

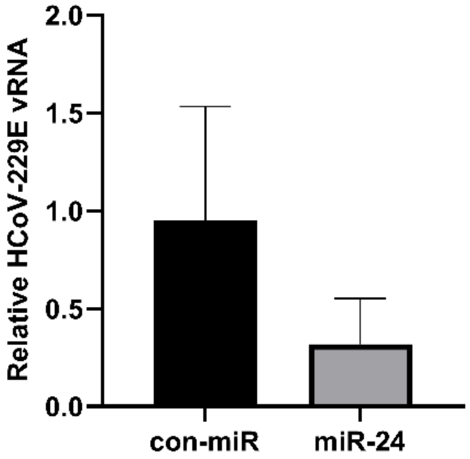

Figure S6. miR-24-3p shows a trending inhibition against Human coronavirus 229E. A549 cells were reverse transfected with miR-24-3p or a control miRNA for 24h before infecting with HCoV-229E, MOI of 0.05, for 48h. Following infection, cells were lysed and RT-qPCR was performed (technical duplicate) for vRNA of HCoV-229E normalized to GAPDH mRNA. n = 2. Error bars represent SEM. p = 0.2907.

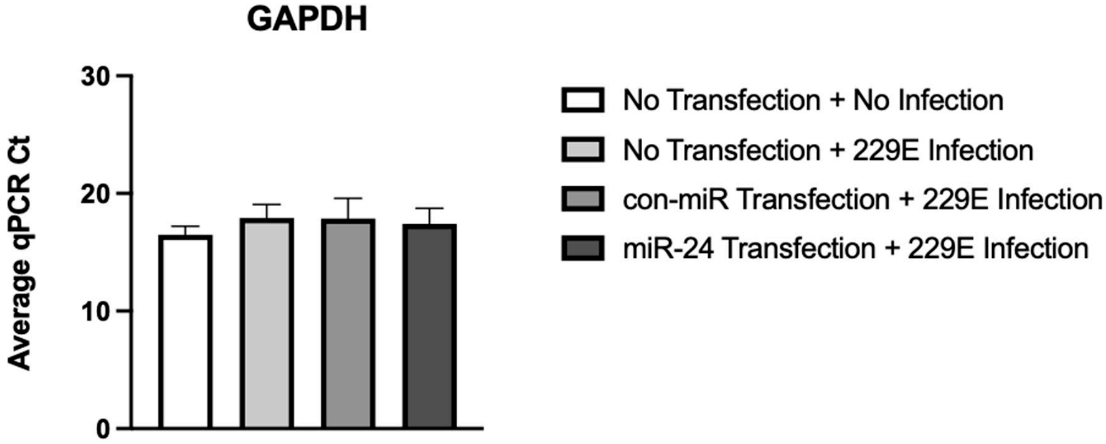

Figure S7. GAPDH is not affected by transfection of miR-24 followed by HCoV-229E infection. A549 cells were reverse transfected with miR-24 or a control miRNA for 24h before infecting with HCoV-229E, MOI of 0.05, for 48h. Following infection, cells were lysed and RT-qPCR was performed in technical duplicate. n = 2. Error bars represent SEM.
